# A Deep Learning-Based Automatic Mosquito Sensing and Control System for Urban Mosquito Habitats

**DOI:** 10.3390/s19122785

**Published:** 2019-06-21

**Authors:** Kyukwang Kim, Jieum Hyun, Hyeongkeun Kim, Hwijoon Lim, Hyun Myung

**Affiliations:** 1 Department of Biological Sciences, Korea Advanced Institute of Science and Technology, 291 Daehak-ro, Daejeon 34141, Korea; kkim0214@kaist.ac.kr; 2 Department of Civil and Environmental Engineering, Korea Advanced Institute of Science and Technology, 291 Daehak-ro, Daejeon 34141, Korea; jimi.hyun@kaist.ac.kr; 3 Department of Mechanical Engineering, Korea Advanced Institute of Science and Technology, 291 Daehak-ro, Daejeon 34141, Korea; hkkim1227@kaist.ac.kr; 4 School of Electrical Engineering, Korea Advanced Institute of Science and Technology, 291 Daehak-ro, Daejeon 34141, Korea; wjuni@kaist.ac.kr

**Keywords:** mosquito, vector control, deep learning, urban habitat, drug spray

## Abstract

Mosquito control is important as mosquitoes are extremely harmful pests that spread various infectious diseases. In this research, we present the preliminary results of an automated system that detects the presence of mosquitoes via image processing using multiple deep learning networks. The Fully Convolutional Network (FCN) and neural network-based regression demonstrated an accuracy of 84%. Meanwhile, the single image classifier demonstrated an accuracy of only 52%. The overall processing time also decreased from 4.64 to 2.47 s compared to the conventional classifying network. After detection, a larvicide made from toxic protein crystals of the *Bacillus thuringiensis* serotype *israelensis* bacteria was injected into static water to stop the proliferation of mosquitoes. This system demonstrates a higher efficiency than hunting adult mosquitos while avoiding damage to other insects.

## 1. Introduction

Mosquitoes annually cause significant damage to mankind as they disseminate various deadly infectious diseases including malaria, yellow fever, or encephalitis [1]. The recent outbreak of the Zika virus [2] has again awakened the public to the dangers of mosquitoes and the necessity of vector control. Classic mosquito control approaches are generally focused on the control of the adult mosquito via thermal fogging trucks, pesticides, or even automated electrical mosquito traps [3]. However, the mosquito is a small, flying insect which tends to hide near urban areas. Consequently, pesticides and fogs have difficulty in permeating into these hideouts. Given the impact on the surrounding ecosystem and the need to use these preventive measures near cities, the toxicity of the chemicals is also limited. Mosquito nets represent a preventive measure, but not a fundamental solution, for reducing the proliferation of mosquitoes.

Compared to the imago stage, mosquito larvae tend to proliferate and gather in static water such as puddles. This feature makes the control of mosquito larvae much more efficient than controlling them at the imago stage [4]. The well-known habitats of mosquito larvae are the puddles generated after rainfall. However, recent research has shown that most mosquito habitats found in urban areas are located near rainfall collection wells [5] and sewage tanks located under buildings [6]. These urban/indoor facilities enable mosquitoes to increase in number throughout the year, and not only in summer when rain is frequent.

Many larvicides have been developed to kill mosquito larvae, and one of the most promising larvicides is composed of toxic protein crystals extracted from the spores of the *Bacillus thuringiensis* serotype *israelensis* (Bti) bacteria [4]. The toxicity is species specific and a low rate of tolerance was observed. Although the Bti treatment is effective and eco-friendly, it does not affect a large area and is washed away rapidly (in less than a month). Furthermore, it is impossible to spray Bti larvicide into every candidate puddle or region where mosquito larvae can grow using human labor alone.

Instead of monitoring every water-related facility in a given urban area, we propose an automated mosquito detection/larvicide spray system installed at the mosquito habitat candidate points. Additionally, instead of passive traps (no detection) or a simple object detector [3], which counts the presence of flying insects, we try to obtain static images of the mosquitoes with a camera for image processing-based mosquito sensing.

Recent advances in deep neural network structures represented by the convolutional neural network (CNN) have demonstrated a remarkable increase in performance compared to conventional machine learning methods [7,8,9]. Many applications based on deep learning have been developed, including vermin monitoring such as jellyfish swarm detection [10]. This research has also applied deep learning based-architecture for the image processing. As the importance of the control of mosquitoes has increased, some research on detecting mosquitoes from a single image using neural networks have been conducted [11,12,13].

The proposed system is equipped with a clean, white mosquito observation pad that lures adult mosquitoes. An attached camera records images of the mosquitoes on the pad, attracted by the lures. If the mosquito larvae proliferate at the installed point (a constant or increasing number of mosquitoes is observed), the attached robot activates and drops a Bti larvicide package into the water to exterminate the growing mosquito larvae. Therefore, our proposed system is a novel approach to controlling mosquitoes, which not only uses deep learning-based image classification, but also uses own hardware equipped with a luring pad and Bti dropper. In addition, the whole control system is placed in an indoor experimental site and verifies that it can exterminate the mosquito larvae.

## 2. Materials and Methods

### 2.1. Platform Overview

**Figure 1:**
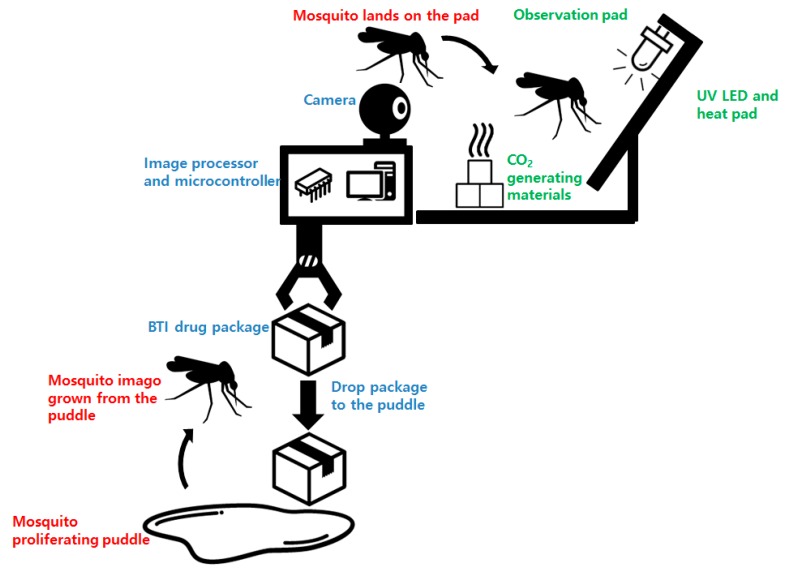
Overall procedure of the proposed mosquito detection and control system.

**Figure 2:**
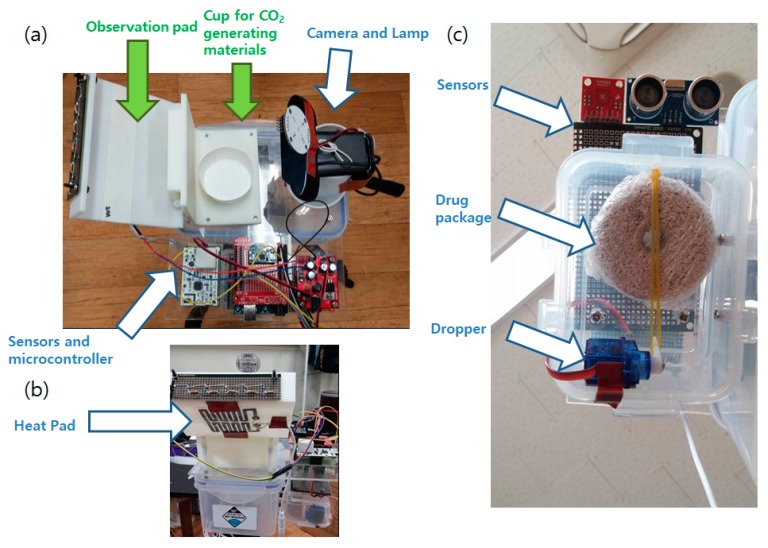
(**a**) The mosquito control system developed. (**b**) Heat pad and Ultra Violet Light-Emitting Diode (UV LED) attached to the mosquito observation pad. (**c**) Close-up view of the automatic Bti larvicide dispenser.

The system is equipped with a mosquito observation pad and a linked camera for mosquito image acquisition. To lure the mosquitoes, an Ultra Violet (UV) Light-Emitting Diode (LED), a 39 °C heat pad, and CO_2_-generating chemicals (sodium hydrogen carbonate and acetic acid) were used [14,15]. Fruit flavor was added to the acetic acid for a better luring effect. Mosquitoes tend to land on nearby walls or structures before approaching the target (the CO_2_ source in this case). The white observation pad was designed to induce mosquito landing and block the background for easier image processing. For the lighting of the observation pad, a white visible light LED lamp equipped with a timer was attached. Streamed images from the webcam are sent to the computer for image processing. The overall structure of the system is shown in Figure 1. The hardware system developed for this purpose is shown in Figure 2.

### 2.2. Mosquito Luring Experiments

The mosquito luring experiment was conducted in the basement of the W2 building at Korea Advanced Institute of Science and Technology (KAIST). The indoor sewage tank was placed in the basement of the building and the dissemination of the mosquitoes was reported. The developed sensor node was placed in the room near the sewage tank. The sensor node was placed in the dark for 15 min and the lamp was turned on and off in a 15 min cycle. The attached camera recorded the image of the observation pad and was subsequently used for the image-based mosquito sensing. As a negative control of the mosquito lure, an experiment with an empty observation pad without CO_2_-generating chemicals was also performed for comparison.

### 2.3. Image Preparation and Deep Learning Architecture

The deep learning architecture-based pipeline was developed for image processing and mosquito detection in the recorded videos. The observation pad attached to the sensor node blocks the background noise and generates a plain background in the video. The grayscale conversion and thresholding were performed to find black objects on the screen. As the distance between the observation pad where the mosquitoes land and the camera is fixed, the size of the mosquitoes generally falls into a certain boundary. The contour in the threshold images was filtered based on their size and saved as 60 × 60 color image patches. The collected patches were manually classified into mosquito and non-mosquito images and the deep learning image classifier was trained to recognize them. AlexNet [7] was originally designed for the ImageNet database [16] classification task with a learning rate of 0.001 and with a step-down decay used for the classification. Of the collected image patches, 25% (approximately 2000 images in total) were used as a test dataset while the rest of the images were used for the training.

Classifying single image patches obtained by color filtering is a feasible design. However, color is not a very robust method even though the observation pads block the background. Also, classifying every dark object in the image requires multiple forward passes of a network, which requires unnecessarily long computing time and resources. Instead, this research applied a fully convolutional network (FCN) [17] and neural network-based regression to count the number of mosquitoes in the image with the least network inference. The summary of the network is shown in Table 1. The total number of parameters in the FCN is approximately 134.82 M.

**Figure 3:**
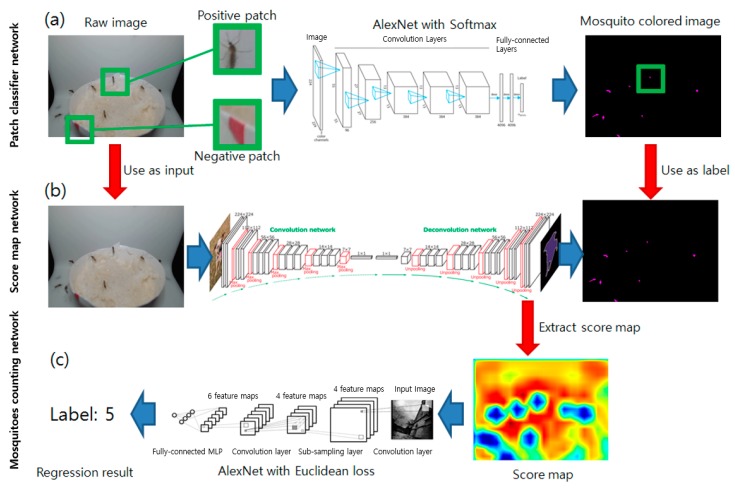
Flow diagram of the deep learning-based mosquito counting pipeline. The red arrows indicate data transfer and the blue arrows indicate network input/output. (**a**) Patch classifier network: mosquito colored-only image preparation using the pre-trained image patch classification network. (**b**) Score map network: FCN training based on the generated label from the previous stage. (**c**) Mosquito counting network: regression using a score map extracted from the trained FCN.

Label image datasets were generated by changing all pixel values to black (0,0,0 in RGB) except the mosquito regions detected by the pre-trained patch classifying network. The regions corresponding to the mosquitoes were changed to purple (255,0,255 in RGB). The FCN network, which infers the possibility that each pixel belongs to the given classes, was used for detecting the mosquito-like regions in the image. After the training, the probability map before the final deconvolution layer is extracted and passed to the regression network. The same AlexNet structure used in the patch classification was leveraged but the loss layer was changed to the Euclidean distance loss from the softmax cross entropy for regression purposes. The number of mosquitoes in the image was used as a label. Labels of 0 to 5 were prepared and numbers larger than 5 were considered as label 5. Approximately 60 images per label were prepared. The overall pipeline process is shown in Figure 3. A Caffe framework [18] with Python binding was used for the implementation of the system. The summary of the network is shown in Table 2. The total number of parameters in AlexNet is approximately 60.97 M.

The score map before the final deconvolution layer was extracted and used as a final output as shown in Figure 3b. The original purpose of the FCN was to obtain the outline of the target object in the image by examining the pixel-wise associability of the image. However, the FCN was not used in this research to detect the exact edge of the mosquitoes in the image; instead, it marked the rough location of the mosquitoes in the image. By doing so, the number of images required to train the FCN was decreased (the FCN requires a calculated probability over a certain threshold to generate a final output edge and many training samples are required to increase pixel-wise certainty); however, a probability map with rough accuracy is still obtainable.

## 3. Results and Discussion

### 3.1. Mosquito Luring Experiment

**Figure 4:**
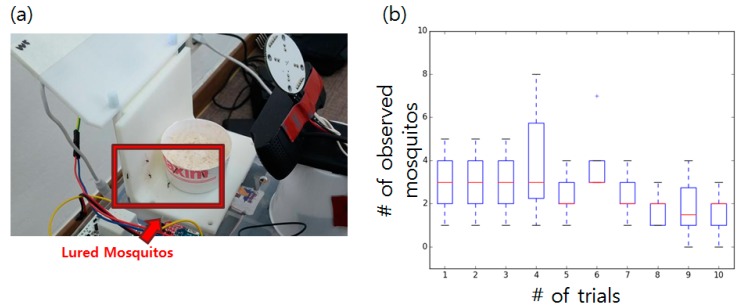
(**a**) Mosquitoes lured by the sensor nodes. (**b**) Boxplot showing the number of mosquitoes observed in the recorded video. Ten random screenshots from 10 videos (trials) were used.

The experiment results demonstrated that the system successfully attracted mosquitoes during its operation. A recorded video showed that the mosquitoes started to approach the trap approximately 14 min after its installation. The number of mosquitoes present at random moments during the 15 min video was manually calculated for statistical purposes. The image of the lured mosquitoes and a boxplot generated with 10 data points from the 10 trials are shown in Figure 4. More than three mosquitoes constantly (median value of three) approached the trap during its operation with a maximum number of eight. The negative control (no luring mechanism used) showed zero or one mosquito during multiple trials. Other insects were not attracted to the observation pad during the luring experiment.

The system is not equipped with an adult mosquito killing or trapping mechanism. UV/visible light-based mosquito traps attract not only mosquitoes, but also useful pollinating insects, thereby causing collateral damage. Although the attracted insects may cause false positive image processing results, only a larvicide drug package is released to avoid killing unwanted insects. Furthermore, the elimination of active traps requiring actuators can help reduce power consumption.

### 3.2. Deep Learning-Based Mosquito Detection

The intermediate deep neural networks required for the procedure introduced in Section 2.3 were developed and trained. Firstly, the AlexNet classifier network for the mosquito/non-mosquito contour classification shown in Figure 3a was trained and examined. The trained network showed an accuracy of 95.25% at the test phase, at the 20th epoch after the training started. Training and test loss started from a value of 0.6 and 0.5 and dropped to 0.15 and 0.2 after all iterations were completed.

**Figure 5:**
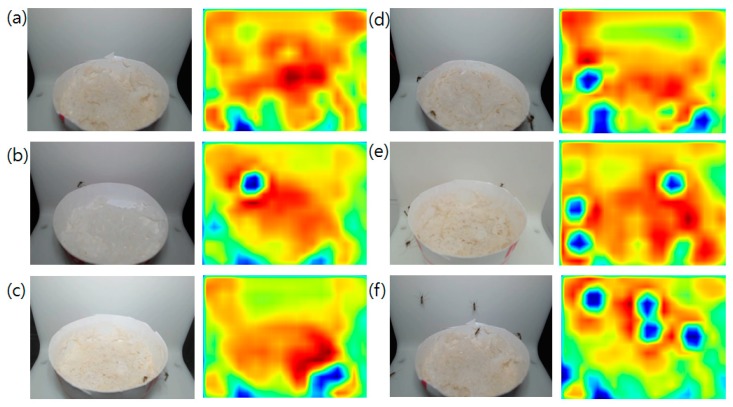
Input images (left) for the FCN and generated score maps (right) showing the probability of pixel matches to the presence of a mosquito. Each label indicates the number of mosquitoes in the image. (**a**) label 0, (**b**) label 1, (**c**) label 2, (**d**) label 3, (**e**) label 4, (**f**) label 5 (this label includes > 5 images as well).

As the training results demonstrated sufficient accuracy in detecting mosquitoes, the prepared network was used to generate mosquito colored-only label images for the FCN training. Approximately 100 images with the corresponding label images were generated and used for the FCN training. The stride size of 32 was used for faster inference time/low memory consumption compared to the other stride size options (16 and 8). The input image and corresponding FCN processed output is shown in Figure 5. The probability that the pixel falls into a region where a mosquito is present is marked with dark blue, while the other less probable regions are colored with a red to green spectrum. The blue dotted region is similarly located at the position where the mosquito exists in the input image. If the system tries to detect the mosquitoes by color-based contour finding and classification, tuning of the thresholding value is required, as it affects the final accuracy of the classifier. Also, multiple inferences, although required, drop the accuracy of the total result and require a certain amount of computation time for each image. By using the FCN, the proposed system can infer the possible number of mosquitoes with a single network inference. However, the generated probability map does not exactly correspond to the mosquito region. Counting the number of blue dots is not applicable as closely-located mosquitoes fall into the same boundary and are marked as a larger single dot. Thus, the generated score maps were passed to the regression network for neural network-based regression to estimate the number of mosquitoes based on the probability map.

### 3.3. Examination of the Mosquito Counting Pipeline

**Figure 6:**
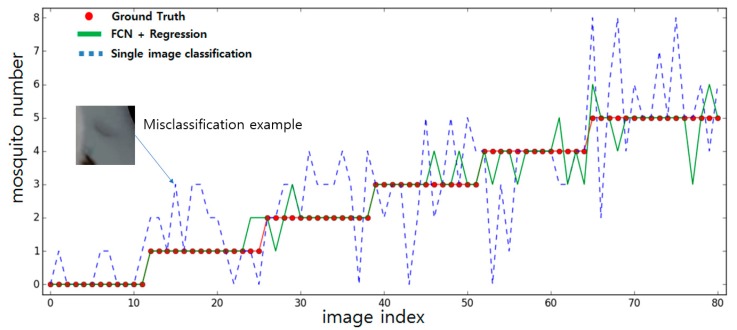
Red circles indicate the ground truth value—the real number of mosquitoes in the image. The green line represents the estimation results from the FCN and neural network-based regression. The blue dotted line shows the number of mosquitoes detected by classifying every contour in the image using the classification network. The example of the misclassification image patch which causes the overestimate of the mosquito detection is represented on the left side.

The generated score maps were trained to the modified AlexNet introduced in Section 2.3. The number of mosquitoes in the image was used as a label for the regression and ground truth for the performance measurement. As a comparison, the image classification network classifying the contour obtained from the image was compared with the FCN and neural network-based regression. The results are shown in Figure 6.

A total of 80 test images were processed and compared by the two networks. The label is limited to 5 as most images have less than 5 mosquitoes. A few images containing more than 5 mosquitoes were also referred to with a label of 5. If the network output exceeded 5 while processing the label 5 image, it was considered as a correct answer. The FCN and neural network-based regression result demonstrated an accuracy of 84%. Meanwhile, the single image classifier demonstrated an accuracy of only 52%. As shown in Figure 6, the blue line indicating the inference results from the classifier network has more divergence from the ground truth (the red dotted line) compared to the results from the regression network (the green line). As previously mentioned, though the trained classifier shows good classification results when classifying a single image patch, giving perfect answers for every image patch is a difficult task. Root mean square errors (RMSEs) of 1.37 (blue) and 0.42 (green) were obtained, showing that the regression network with the FCN score map input has much better performance in number estimation tasks. The proposed FCN and neural network-based regression demonstrated an error of ±1 most of the time, while the classification network had errors within a much higher range.

Although it is possible to count each mosquito, it is necessary to measure a level exceeding a certain number rather than an exact number of vermin when the government notifies alarms about them, such as jellyfish counting in the South Korea [19]. Also, in order to obtain an accurate outline in the FCN, it is necessary to have many training sets. It is possible to implement the desired function with a relatively small amount of data if the FCN is only trained enough to generate a good probability map instead of the outline proposal and combined with the regression network.

The processing time required for the inference was also decreased. For the processing of 80 images, the proposed network required 2.47 s on a platform with an Intel i7-6700K CPU and 2 Nvidia GTX 1080 GPUs. The conventional classification network took 4.64 s to process approximately 200 mosquitoes in the 80 images. Though the depth of the network is deeper with the FCN and neural network-based regression, two inferences require much less time when compared to multiple AlexNet inferences.

The proposed system is aimed at optimizing network usage for operation in embedded systems. Therefore, lighter network structures are preferred. The effort and human labor required to generate training data are also decreased by using the proposed pipeline as compared to the object detection network which requires the exact boxing of every mosquito in the image.

## 4. Conclusions and Future Work

This paper presents the development of an automated mosquito detection and control system based on the image processing of lured mosquitoes. The system can detect mosquitoes and release a Bti drug package into a target area, for example, sewage, if it is considered to be a mosquito larvae habitat. Thus, control of the mosquito population is more efficient than only removing mosquitoes at the imago stage. We expect that this system could be used not only in indoor urban areas, but also at rainfall collection facilities found in the tropical regions of developing countries.

In the repeated experiment, we could not find insects other than mosquitoes in our experimental site. However, for generality, we need to distinguish mosquitoes from any other kinds of insects which can be found outdoors. This will be a future task of our research.

As part of future research, we are considering optimizing the multiple network stacked structure. We are also considering benchmarking end-to-end structures and recent YOLO networks [20,21,22] to increase the object detection performance. Replacing AlexNet with ResNet to make the system faster is being considered. In addition, attaching the sensor node to a mobile system, such as a drone, is also being considered.

## Figures and Tables

**Table 1:** A summary of the proposed network architecture based on the Fully Convolutional Network (FCN).

Name	Type	Input Size	Output Size	Kernel Size	Stride	# of Filters
data	data	3 × 500 × 500	3 × 500 × 500			
conv1	convolution	3 × 500 × 500	64 × 500 × 500	3		2
pool1	max pooling	64 × 500 × 500	64 × 250 × 250	2	2	1
conv2	convolution	128 × 250 × 250	128 × 250 × 250	3		2
pool2	max pooling	128 × 250 × 250	128 × 125 × 125	2	2	1
conv3	convolution	256 × 125 × 125	256 × 125 × 125	3		3
pool3	max pooling	256 × 125 × 125	256 × 63 × 63	2	2	1
conv4	convolution	512 × 63 × 63	512 × 63 × 63	3		3
pool4	max pooling	512 × 63 × 63	512 × 32 × 32	2	2	1
conv5	convolution	512 × 32 × 32	512 × 32 × 32	3		3
pool5	max pooling	512 × 32 × 32	512 × 16 × 16	2	2	1
fc6	convolution	512 × 16 × 16	4096 × 10 × 10	7		1
drop6	dropout (rate 0.5)	4096 × 10 × 10	4096 × 10 × 10			
fc7	convolution	4096 × 10 × 10	4096 × 10 × 10	1		1
drop7	dropout (rate 0.5)	4096 × 10 × 10	4096 × 10 × 10			
score	convolution	4096 × 10 × 10	21 × 10 × 10	1		1
score2	deconvolution	21 × 10 × 10	21 × 22 × 22	4	2	1
score-pool4	convolution	512 × 32 × 32	21 × 32 × 32	1		1
score-pool4c	crop	21 × 32 × 32	21 × 22 × 22			
score-fuse	eltwise	21 × 22 × 22	21 × 22 × 22			
bigscore	deconvolution	21 × 22 × 22	21 × 368 × 368	32	16	1
upscore	crop	21 × 368 × 368	21 × 500 × 500			
output	softmax	21 × 500 × 500	21 × 500 × 500			

**Table 2:** A summary of the proposed network architecture based on AlexNet (LRN: Local Response Normalization).

Name	Type	Input Size	Output Size	Kernel Size	Stride	# of Filters
data	data	3 × 227 × 227	3 × 227 × 227			
conv1	convolution	3 × 227 × 227	96 × 55 × 55	11	4	1
norm1	LRN	96 × 55 × 55	96 × 55 × 55			
pool1	max pooling	96 × 55 × 55	96 × 27 × 27	3	2	1
conv2	convolution	96 × 27 × 27	256 × 27 × 27	5		1
norm2	LRN	256 × 27 × 27	256 × 27 × 27			
pool2	max pooling	256 × 27 × 27	256 × 13 × 13	3	2	1
conv3	convolution	256 × 13 × 13	384 × 13 × 13	3		1
conv4	convolution	384 × 13 × 13	384 × 13 × 13	3		1
conv5	convolution	384 × 13 × 13	256 × 13 × 13	3		1
pool5	max pooling	256 × 13 × 13	256 × 6 × 6	3	2	1
fc6	InnerProduct	256 × 6 × 6	4096 × 1 × 1			1
drop6	dropout (rate 0.5)	4096 × 1 × 1	4096 × 1 × 1			
fc7	InnerProduct	4096 × 1 × 1	4096 × 1 × 1			1
drop7	dropout (rate 0.5)	4096 × 1 × 1	4096 × 1 × 1			
fc8	InnerProduct	4096 × 1 × 1	1000 × 1 × 1			1
loss	SoftmaxWithLoss	1000 × 1 × 1	1000 × 1 × 1			
